# *Bacillus licheniformis* JF-22 to Control *Meloidogyne incognita* and Its Effect on Tomato Rhizosphere Microbial Community

**DOI:** 10.3389/fmicb.2022.863341

**Published:** 2022-04-07

**Authors:** Jianfeng Du, Qixiong Gao, Chao Ji, Xin Song, Yue Liu, Huying Li, Chaohui Li, Pengcheng Zhang, Jintai Li, Xunli Liu

**Affiliations:** ^1^College of Forestry, Shandong Agriculture University, Taian, China; ^2^Key Laboratory of National Forestry and Grassland Administration on Silviculture of the Lower Yellow River, Shandong Agricultural University, Taian, China; ^3^College of Plant Protection, Shandong Agricultural University, Taian, China; ^4^College of Biological and Agricultural Engineering, Weifang University, Weifang, China

**Keywords:** *Bacillus licheniformis*, microbial community, volatile substances, *Meloidogyne incognita*, microbial community composition

## Abstract

*Meloidogyne incognita* is one of the most destructive soil pests, causing serious economic losses in tomato production. Here, *in vitro* experiments demonstrated that the *Bacillus licheniformis* strain JF-22 has the potential to prevent *M. incognita* infection. A pot experiment confirmed that *B. licheniformis* strain JF-22 isolated from the tomato rhizosphere soil and planted in the tomato root-knot nematode disease area effectively prevented and controlled *M. incognita*, reducing its negative effect on tomato growth. Additionally, the composition of volatile substances secreted by *B. licheniformis* strain JF-22 was analyzed using solid-phase microextraction and gas chromatography–mass spectrometry. We detected acetoin, 2,3-Butanediol, [R-(R*,R*) ]-, and hexamethyl cyclotrisiloxane as the main components among these volatiles. Using MiSeq sequencing technology and bioinformatics, we analyzed the influence of *B. licheniformis* strain JF-22 on the microbial community of the tomato rhizosphere. *B. licheniformis* strain JF-22 changed the composition of the microbial community; particularly, it significantly reduced the diversity of the fungal community. Furthermore, using the FUNGuild and PICRUSt databases, we predicted the effect of JF-22 on microbial community function. In conclusion, *B. licheniformis* strain JF-22 may be considered as a potential biocontrol agent against *M. incognita*.

## Introduction

The tomato root-knot nematode disease caused by *Meloidogyne incognita* is a devastating disease that affects tomatoes (Siddiqui et al., 2006). *M. incognita* absorbs key nutrients after infecting plant roots and can easily induce secondary infections by a range of soil pathogens, thereby negatively affecting the plant growth and, ultimately, yield. *M. incognita* is transmitted *via* the soil, water sources, and diseased plants (Roberts et al., 1981). Specifically, under greenhouse conditions, the disease may occur continuously because of repeated cropping. Moreover, it has developed into a disaster in some large vegetable-growing areas (Fravel, 2005).

Plant growth-promoting rhizobacteria (PGPR) are an important biological resource (Bhattacharyya and Jha, 2012). Studies have shown that many PGPR, such as *Bacillus subtilis*, *Bacillus megaterium*, *Bacillus coagulans*, and *Pseudomonas fluorescens*, can control *M. incognita* (Xiang et al., 2018). Indeed, PGPR can reportedly induce plant resistance to *M. incognita* (Siddiqui and Shaukat, 2002). For example, rhizobacterial consortia can be used to prevent parasitic nematodes in grapevines (Aballay et al., 2020). Similarly, *Phanerochaete chrysosporium* can inhibit the growth of J2s (second stage juveniles) of *M. incognita* (Du et al., 2020). Furthermore, studies have shown that *Bacillus* was the main genus responsible for the higher mortality of *M. incognita* (Xiang et al., 2017). *Bacillus* not only directly stimulates the plant growth by enhancing nutrient acquisition or stimulating the host-plant defense mechanism; but, additionally, it inhibits the growth of pathogenic microorganisms (Kumar et al., 2001). For example, *Bacillus halotolerans, Bacillus kochii, Bacillus oceanisediminis, Bacillus pumilus, Bacillus toyonensis, Bacillus cereus, Pseudomonas aeruginosa*, and *Bacillus pseudomycoides* can control the *M. incognita* (Liu et al., 2020). Further, single or multiple rhizosphere bacteria can control the root-knot nematodes. For example, the combination of *Bacillus amyloliquefaciens* and *B. subtilis* strains can reduce the number of *M. incognita* specimens in the soil (Burkett et al., 2008).

The control strategies for *M. incognita* mainly include biological and chemical methods. However, currently, chemical control methods are predominantly in use for the control of plant-parasitic nematodes. Chemical agents have been used for a long time because of their rapid and obvious control effects. However, this method can lead to problems such as environmental pollution and drug resistance in *M. incognita*. Therefore, biological control is now receiving increasing attention (Zhang et al., 2022), particularly because the social awareness of the urgency for environmental protection is increasing. Rhizosphere microorganisms interact with phytopathogens, other indigenous microorganisms, and host plants by competing for nutrients, producing antimicrobial substances, and secreting volatile organic compounds to better adapt to the rhizosphere environment. While doing so, rhizosphere microorganisms perform a disease-suppressing function (Hinsinger et al., 2009). In this study, strain JF-22 of *Bacillus licheniformis* was isolated from the rhizosphere soil in an area where healthy tomato plants were grown in the presence of tomato root-knot nematode. The effect of *B. licheniformis* strain JF-22 on *M. incognita* was studied using *in vitro* tests and pot experiments. Solid phase microextraction (SPME), gas chromatography–mass spectrometry (GC–MS), MiSeq sequencing technology, and bioinformatics analytical methods were used to analyze the composition of volatile substances produced by strain JF-22 and the influence of strain JF-22 on the structure of the rhizosphere microbial community.

## Materials and Methods

### Isolation and Screening of Bacterial Strains

We followed the method described by Wang et al. (2021) to isolate the soil bacteria. Rhizosphere soil in which healthy tomato (*Lycopersicon esculentum*) plants grew was collected from areas infested with tomato root-knot nematode disease. Soil samples (5 g) were suspended in 50 ml of sterile distilled water and mixed on a table concentrator for 30 min. These soil samples were serially diluted (up to 10^–7^-fold), plated on potato dextrose agar (PDA), and incubated at 30 ± 2°C for 2–3 days. Bacterial colonies growing on the plates were isolated according to their morphological characteristics for further study. Subsequently, the isolated strains were screened using *in vitro* tests, for which purpose, they were transferred to an LB solid medium plate. After activation at 30°C, purified single colonies were picked using a sterile inoculating loop and placed in a 250 ml conical flask containing 50 ml LB liquid medium. After 24 h of cultivation at 30°C, with stirring at 200 rpm, the inoculum was connected to the 50 ml LB culture medium at a ratio of 2% and cultured at 30°C and 200 rpm in a 250 ml conical flask for 48 h to prepare a fermentation broth. This broth was then centrifuged at 13,000 rpm for 10 min and filtered through a 0.22 μm sterile syringe filter. Lastly, 0.8 ml of the filtrates was placed in 1.5 ml centrifuge tubes before adding 100 s-stage juveniles (J2s) of *M. incognita* to each centrifuge tube. The corrected mortality of the J2s of *M. incognita* was calculated at 24 h and again at 48 h. Each treatment was repeated six times.

### Identification of JF-22

Strain JF-22 of *B. licheniformis* was preliminarily identified based on its morphological characteristics and then inoculated on PDA medium and observed for colony morphological characteristics after incubation at 32°C for 5 days. The strain was subsequently cultured for 24 h in LB liquid medium prior to Gram staining. Morphology was observed and photographed under a microscope. Further identification of JF-22 was achieved through the analysis of its 16S rDNA and gyrB gene sequences. The DNA of strain JF-22 was extracted with the Tiangen bacterial DNA extraction kit and stored at 4°C. 16S rDNA was amplified by the polymerase chain reaction (PCR) using the bacterial universal primers 27F (5′-AGAGTTTGATCCTGGCTCAG-3′) and 1492R (5′-GGTTACCTTGTTACGACTT-3′). The gyrB gene was amplified by PCR using specific primers G3F (5′-GAAGTCATCATGACCGTTCTGCAYGCNGGNGGNAARTTY GA-3′) and G3L(5′-AGCAGGGTACGGATGTGCGAGCCRTCN ACRTCNGCRTCNGTCAT-3′). The reaction conditions for 16S rDNA amplification were as follows: 95°C for 5 min; 33 cycles at 95°C for 1 min, at 58°C for 30 s, and at 72°C for 2 min, and a final extension at 72°C for 10 min. The reaction conditions of *gyrB* gene amplification were as follows: 96°C for 5 min, 32 cycles at 96°C for 30 s, at 59°C for 30 s, and at 72°C for 1 min, and a final extension at 72°C for 10 min. Then, the PCR products were purified and sequenced by a commercial sequencing company (Sangon Biotech, Shanghai, China). Nucleotide sequences were analyzed and compared with sequences in the GenBank database using BLAST. A phylogenetic tree of the JF-22 strain was constructed with *16S rDNA* and *gyrB* gene sequences using the neighbor-joining method in Mega 7.0.21.

### Pot Test

*Meloidogyne incognita* was isolated and cultured according to the method described by Jing et al. (2020). Tomato seeds of the tested variety were sown in a nursery tray, and when the seedlings reached the fourth true-leaf stage they were transplanted into a plastic pot 20 cm in diameter and infested with 300 J2s specimens of *M. incognita* per plant. Two days after transplanting, each tomato plant was watered with 100 ml of JF-22 bacterial suspension at a concentration of 10^8^ CFU/ml, and the same amount of sterile water was used as a control (CK). After inoculation, pots were randomly placed on the operating platform of a greenhouse. Each treatment was repeated 60 times. Tomato plants were uprooted separately to determine plant biomass indices. Disease severity was determined using the method of Lu et al. (2017), and the incidence index was determined. Rhizosphere soil around the tomato roots was collected and stored at −80°C for analysis of microbial community structure.


$$\text{Disease}\,\mathrm{severity}\,\mathrm{index}=\frac{\Sigma \,\mathrm{rank}\times \mathrm{no}.\mathrm{of}\,\mathrm{plants}\,\mathrm{in}\,\mathrm{rank}}{\mathrm{Total}\,\mathrm{no}.\mathrm{of}\,\mathrm{plants}\times \mathrm{Maximum}\,\mathrm{rank}}\times 100\%$$

### Analysis of Volatile Organic Compounds by Gas Chromatography–Mass Spectrometry

The volatile composition of strain JF-22 was determined using SPME–GC–MS as described by Wang et al. (2021). Strain JF-22 was inoculated on PDA slants placed in triangular flasks, which were then sealed with foil and incubated at 30°C. After 3 days, polydimethylsiloxane-divinylbenzene (65 μm) was used to collect volatile organic compounds (VOCs), and an RTX-5MS capillary column (60 × 0⋅25 μm ID × 0⋅25-μm thick film) was used to separate VOCs. Helium was used as the carrier gas at a rate of 1 ml min^–1^ to analyze VOCs by GC–MS using the following procedure: desorb SPME-DVB fiber at 200°C for 3 min. For VOCs analysis, first, the oven temperature was raised to 40°C for 3 min, and then increased to 160°C at a rate of 8°C min^–1^; it was maintained at that point for 2 min, and finally increased to 240°C at a rate of 15°C min^–1^ and maintained at that point for 3 min. The mass spectrometer was set to 70 eV electron ionization mode, with a source temperature of 200°C. Continuous scanning from 45 to 500 m/z was used. VOCs were identified by comparison with data provided in the National Institute of Standards and Technology guidelines (number 14).

### DNA Extraction and Illumina MiSeq High-Throughput Sequencing

Total DNA extraction from the soil was performed using the BIO-TEK OMEGA Soil DNA Kit (Omega Bio-tek, Norcross, GA, United States). Concomitantly, the bacterial *16S rDNA* V3-V4 region and the fungal *rDNA-ITS* gene were amplified. PCR amplification was performed as previously described (Sui et al., 2019). After mixing the PCR products of the same sample, they were separated using 2% agarose gels, and the recovered products were purified using AxyPrep DNA Gel Extraction Kit (Axygen Biosciences, Union City, CA, United States), and detected by 2% agarose gel electrophoresis. The recovered products were quantified using a Quantus fluorometer (Promega, United States). Furthermore, the NEXTFLEX Rapid DNA-Seq Kit was used for the DNA library construction. The library was constructed following these steps: (1) Adapter linking; (2) Magnetic bead screening to remove adapter self-ligating fragments; (3) PCR amplification to enrich library templates; (4) Magnetic beads recovery of PCR products to produce the final library. Sequencing was performed on the Illumina MiSeq PE300 platform. The raw sequences were quality controlled using the Trimmomatic software and spliced using the FLASH software as follows: (1) the 300 bp reads were truncated at any site receiving an average quality score of < 20 over a 50 bp sliding window, and the truncated reads shorter than 50 bp were discarded, reads containing ambiguous characters were also discarded; (2) according to the overlap relationship between PE reads, paired reads were merged into a sequence, and the minimum overlap length was 10 bp; (3) maximum mismatch ratio allowed in the overlap region of the spliced sequence was 0.2, and the non-conforming sequences were screened; and (4) samples were distinguished according to the barcodes and primers at the beginning and end of the sequence, and the sequence direction was adjusted. The number of mismatches allowed by the barcode was zero, and the maximum number of primer mismatches was 2. All reads were clustered with a 97% similarity cut-off using UPARSE (ver. 7.1)^1^, and chimeric sequences were identified and removed using UCHIME (Zheng et al., 2015). The taxonomy of each *16S rDNA* and *ITS rDNA* gene sequence was analyzed using the RDP Classifier against the Silva (SSU123) *16S rDNA* database (Amato et al., 2013) and the UNITE 7.0/ITS database (Abarenkov et al., 2010), respectively, using a confidence threshold of 70%. Bacterial population functions were analyzed using the PICRUSt 2 database. Fungal ecosystem analyses were performed using the FUNGuild database (Nguyen et al., 2015). Raw reads were deposited in the NCBI Sequence Read Archive database (accession numbers: PRJNA803317 and PRJNA803323).

### Statistical Analysis

Data were analyzed using Microsoft Excel and SPSS 22.0 (SPSS Inc., Chicago, IL, United States). Duncan’s multi-range test and Student’s *t*-test were performed to compare the significance of the differences at *p* < 0.05.

## Results

### Isolation and Screening of Bacterial Strains

For the selection of potential biocontrol agents against *M. incognita*, 15 bacterial strains were isolated, purified, and screened using *in vitro* assays to test the toxicity of the isolated fermentation supernatant on J2s of *M. incognita*. The results showed that the fermentation supernatant of four bacterial strains had toxic effects on *M. incognita* J2s. Among these, specifically, the fermentation supernatant of JF-22 had the strongest toxic effect on *M. incognita* J2s. When J2s of *M. incognita* were treated for 24 h with the fermentation supernatant of JF-22, the death rate of the J2s was 77%, and when they were treated for 48 h, mortality reached 89%. This indicated that JF-22 has a strong ability to kill *M. incognita* at the J2s developmental stage (Table 1).

**TABLE 1:** Ability of JF-22 to kill *Meloidogyne incognita* at the J2s stage.

Project	JF-22	JF-17	JF-21	JF-26
24 hour	77% ± 1.2a	26% ± 1.6a	42% ± 0.4a	40% ± 1.9a
48 hour	89% ± 0.6b	36% ± 2.0b	44% ± 0.3b	43% ± 0.8b

*24 and 48 h represent corrected mortality of J2s of Meloidogyne incognita at 24 and 48 h after treatment initiation. Different lowercase letters within columns indicate significant differences (p < 0.05) according to Duncan’s multiple range test.*

### Identification of *B. licheniformis* JF-22

**FIGURE 1:**
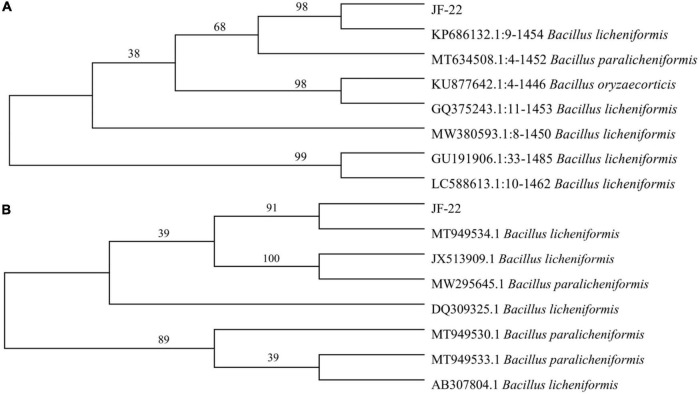
Phylogenetic tree shows relationship between JF-22 and other strains. **(A)** Phylogenetic tree constructed based on *16S rDNA* sequences of strain JF-22 and 7 other *Bacillus* strains; **(B)** Phylogenetic tree constructed based on *gyrB* gene sequences of strain JF-22 and 7 other *Bacillus* strains. Bootstrap values (%) presented at the branches were calculated from 1,000 replications.

The colony of strain JF-22 was round, white, and non-transparent, with a wrinkled surface, irregular edges, and a central bulge. Under the microscope, strain JF-22 was found to be a rod-shaped, gram-positive bacterium. Based on phylogenetic analysis of *16S rDNA* and *gyrB* (Figure 1A), strain JF-22 was identified as *B. licheniformis* (Figure 1B).

### Effects of JF-22 on Plant Growth and Development

The biological functions of *B. licheniformis* strain JF-22 were verified by a pot test. Compared with the control treatment CK, JF-22 significantly increased tomato biomass by 18.41%. The application of *B. licheniformis* strain JF-22 significantly reduced the severity of the root-nematode disease, indicating that *B. licheniformis* effectively controlled *M. incognita* (Table 2).

**TABLE 2:** Effects of *Bacillus licheniformis* strain JF-22 on tomato plant growth and root-nematode disease severity.

Treatment	CK	JF-22
The disease index:%	0.87 ± 0.02a	0.63 ± 0.04b
Biomass (g)	11.19 ± 0.37b	13.25 ± 0.66a

*Different lowercase letters within columns indicate significant difference (p < 0.05).*

### Analysis of Volatile Organic Compounds by Gas Chromatography–Mass Spectrometry

**FIGURE 2:**
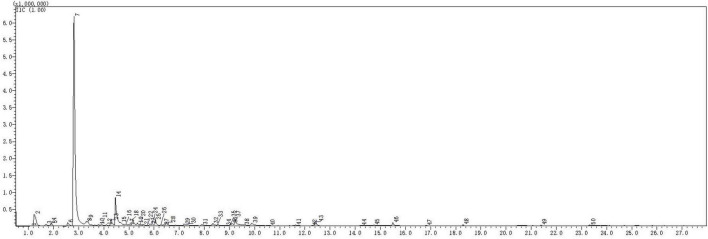
Gas chromatography–mass spectrometry (GC–MS) chromatogram of volatile organic compounds (VOCs) produced by JF-22 strain.

The types and relative contents of VOCs produced by the JF-22 fraction were analyzed using SPME–GC–MS. Figure 2 and Table 3 show SPME–GC–MS results for VOCs produced by JF-22 strain. Volatiles produced by JF-22 were identified using NIST17 and NIST17s (National Institute of Standards and Technology) standard mass spectrometry libraries. Among VOCs produced by JF-22, acetoin and 2,3-Butanediol, [R-(R*,R*)]- were the main components, with the relative peak areas of 64.2 and 7.89%, respectively, followed by cyclotrisiloxane, hexamethyl-; cyclotetrasiloxane, octamethyl-; 3-Hexanol, 2-methyl-; cyclopentasiloxane, decamethyl-; and 3-Pentanol and silanediol, dimethyl-.

**TABLE 3:** The GC–MS of major components in VOCs produced by JF-22 strain.

Components	Rt (min)	Area (%)
Silanediol, dimethyl-	2.565	1.04
Acetoin	2.813	64.2
2,3-Butanediol, [R-(R*,R*)]-	4.461	7.89
3-Pentanol	4.667	1.12
Cyclotrisiloxane, hexamethyl-	5.15	1.89
3-Hexanol, 2-methyl-	6.05	1.41
Cyclotetrasiloxane, octamethyl-	9.223	1.73
Cyclopentasiloxane, decamethyl-	12.392	1.34

The 2,3-Butanediol, [R-(R*,R*)]- is [R,R]-2,3-butanediol; (Z)-2,3-butanediol; (R,R)-(-)-butane-2,3-diol (CAS Registry Number: 24347-58-8).

### Analysis of Data Characteristic of Soil Samples and Microbial Community Diversity

After sequence optimization, there were 252,803 sequences in the two treatment groups, with an average of 42,134 in each soil sample of bacteria. And there were a total of 234,239 ITS sequences, with an average of 39,040 in each soil sample. The sequence generated by sequencing was clustered and segmented at a similarity level of 97%, and corresponding bacterial and fungal operational taxonomic units (OTUs) were generated, respectively. The bacterial community structure diversity indexes Shannon and Chao in the CK and JF-22 groups didn’t change significantly. However, in the richness index of fungal community, the fungal diversity indexes Shannon and Chao of the JF-22 group decreased significantly compared to CK (Table 4).

**TABLE 4:** Diversity and richness indices of microbial communities from the CK and JF-22 experimental groups.

Index	Bacteria		Fungi	
	CK	JF-22	CK	JF-22
Shannon	6.19 ± 0.12	6.24 ± 0.09	3.19 ± 0.25	2.32 ± 0.30*
Chao	2770.27 ± 95.25	2754.36 ± 33.29	387.87 ± 38.52	229.83 ± 25.34**

*, significant difference (p < 0.05); **, highly significant difference (p < 0.001); Chao, Chao1 richness estimator; Shannon, Shannon–Wiener diversity index.

### Comparative Analysis of Microbial Community Composition Between JF-22-Treated and Untreated Soils

**FIGURE 3:**
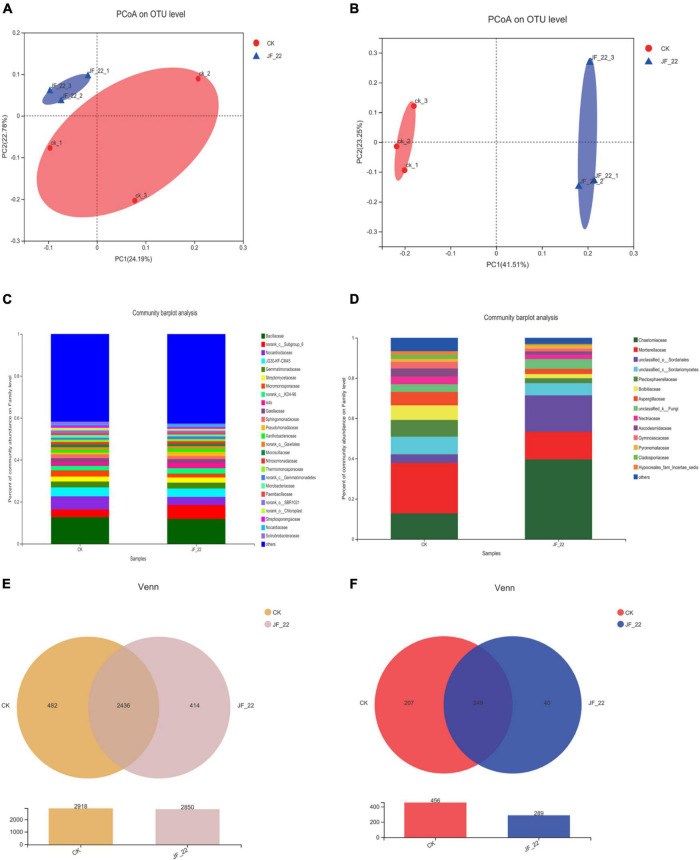
The effect of JF-22 to tomato rhizosphere soil microbial community. **(A)** PCoA analysis based on the unweighted unifrac distances of bacterial community. **(B)** PCoA analysis based on unweighted unifrac distances of fungal community. **(C)** The relative abundance in family of tomato rhizosphere bacterial. **(D)** The relative abundance in family of tomato rhizosphere fungi. **(E)** Venn diagram of OTUs between CK- and JF-22 relate bacterial; **(F)** Venn diagram of OTUs between CK and JF-22 relate fungi.

Using high-throughput sequencing, we analyzed the effects of JF-22 inoculants on the tomato–rhizosphere microbial communities. PCoA analysis based on unweighted UniFrac distance analysis showed that *B*. *licheniformis* strain JF-22 changed the composition of the microbial community in the tomato rhizosphere, particularly, that of the fungal communities (Figures 3A,B). Further, the bacterial community in the tomato rhizosphere soil across treatments was mainly composed of 25 families whose members were the same across treatments, although the relative abundance of each member differed. Thus, for example, abundance of Norank_c_Subgroup_6 and *Nocardioidaceae* differed greatly among treatments. In the CK treatment, the relative abundance of norank_c_Subgroup_6 was 3.6%, but in the JF-22-treated pots, the relative abundance of norank_c_Subgroup_6 was 6.6%, and the relative abundance of *Nocardioidaceae* was 6.3, and 3.9% in the CK and the JF-22 treatment groups, respectively (Figure 3C).

The fungal community in the tomato rhizosphere was mainly composed of 14 families across treatments. The overall composition of the rhizosphere fungal communities was similar in all treatments, but the abundance of each member was different. Specifically, Chaetomiaceae showed the largest difference between treatments. In CK pots, the relative abundance of Chaetomiaceae was 12.6%, while it was 39.7% in JF-22-treated pots. Similarly, Mortierellaceae and unclassified _o_Sordariales differed between treatments. In CK, the relative abundance of Mortierellaceae was 25.2%, while it was 13.6% in JF-22-treated pots. Furthermore, the relative abundance of unclassified _o_Sordariales was 4.3 and 18.2% in the CK and JF-22-treated pots, respectively (Figure 3D).

The number of unique and common OTUs in the multiple samples is shown in a Venn diagram. In the case of bacteria, 2,918 OTUs were identified in CK and 2,850 OTUs were found in JF-22, among which, 2,436 were common to both CK and JF-22-treated pots, while 482 were unique to CK and 414 to JF-22-treated pots (Figure 3E). As for fungi, 456 OTUs were identified in CK and 289 in JF-22-treated pots. Among them, 249 were common to both CK and JF-22-treated pots (Figure 3F). These findings indicated that the application of JF-22 can change the composition of the tomato rhizosphere–soil microbial community.

### Prediction of Rhizosphere Microbial-Community Function

**FIGURE 4:**
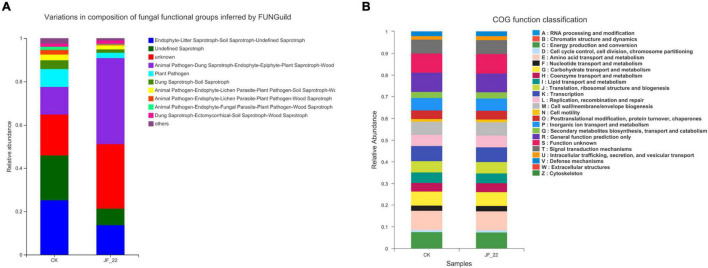
Analysis of the function of the rhizosphere microbial community. **(A)** COG function classification analysis, and **(B)** FUNGuide functional classification statistical histogram.

FUNGuild was used to analyze the functional guild annotation of the fungi present across treatments. In the CK group, the number of plant pathogens was 2,516, and the number of Dung Saprotrophs and Soil Saprotrophs was 1,221; meanwhile, in the JF-22 group, the number of plant pathogens was 739, and the number of Dung Saprotrophs and Soil Saprotrophs was 737, indicating that *B. licheniformis* strain JF-22 may reduce the number of plant pathogens, pathogenic bacteria, dung saprotrophs, and soil saprotrophs (Figure 4A). Compared to the results of FUNGuild, the results of the CoG function classification did not differ much between the experimental treatment groups (Figure 4B). All samples were rich in energy production and conversion (relative abundance 7.41–7.46%); amino acid transport and metabolism (8.85–8.95%); nucleotide transport and metabolism (2.36–2.40%); carbohydrate transport and metabolism (6.31–6.37%); coenzyme transport and metabolism (4.12–4.13%); lipid transport and metabolism (4.63–4.81%); translation, ribosomal structure, and biogenesis (5.13–5.14%); transcription (6.82–6.96%); replication recombination and repair (5.40–5.46%); cell wall/membrane/envelope biogenesis (5.98–6.14%); post-translational modification, protein turnover, chaperones (3.96–4.00%); inorganic ion transport and metabolism (5.69–5.69%); secondary metabolites biosynthesis, transport, and catabolism (2.69–2.79%); general function prediction only (8.71–8.74%); and signal transduction mechanisms (6.35–6.50%). These COG functional classification results indicate that the microbial communities of tomato rhizosphere–soil bacteria maintained similar biological functions in all treatment groups.

## Discussion

Plant growth-promoting rhizobacteria are important and beneficial rhizosphere microorganisms (Babalola, 2010). The volatile substances produced by PGPR can reduce the incidence of root-knot nematode disease by poisoning nematodes and *via* other mechanisms (Siddiqui and Mahmood, 1999). *B. cereus*, *B. licheniformis*, *Lactobacillus sphaeroides*, *P. fluorescens*, and *P. brassicae* were used in a greenhouse test against *M. incognita* infection, and the results showed that *B. licheniformis* and *P. fluorescens* significantly reduced the infection of tomato roots by second-stage juveniles of *M. incognita* (Colagiero et al., 2017). The endophytic bacterium *B. cereus* BCM2 can affect the rhizosphere secretions (2,4-di-tert-butylphenol, 3,3-dimethyloctane, and n-tridecane), thereby inhibiting the second-stage juveniles of *M. incognita* and reducing the number of nematode specimens in the soil (Li et al., 2019). Similarly, *Bacillus aryabhattai* A08 can reduce the number of *M. incognita* specimens in the soil (Viljoen et al., 2019). As for the experiments reported herein, our data clearly demonstrated a toxic effect of *B. licheniformis* JF-22 on *M. incognita*. Specifically, our pot experiment showed that the JF-22 strain of *B. licheniformis* effectively prevented infection by *M. incognita* and alleviated the negative effects of *M. incognita* infection on tomato growth. Beneficial microorganisms can effectively prevent soil-borne diseases through the secretion of metabolites. For example, *Bacillus velezensis* FZB42 significantly inhibited the plant pathogen *Ralstonia solanacearum* by secreting benzaldehyde and 1,3-butadiene (Borriss et al., 2019).

This study analyzed the types of VOCs produced by JF-22 using SPME–GC–MS and found many ingredients, such as cyclotrisiloxane and hexamethyl- which reportedly can be toxic to the root-knot *Meloidogyne* (Hamouda, 2013). Further, acetoin can produce 2,3-Butanediol *via* a series of enzymatic reactions (Cui et al., 2021). In turn, 2,3-Butanediol can induce the stomatal closure under stress conditions. Additionally, it induces plant system tolerance; affects salicylic acid, jasmonic acid, and ethylene signaling channels; and promotes plant growth by regulating the expression of genes related to cell wall structure (Yi et al., 2016; Wu et al., 2018). Consistently, the application of *Sphingomonas* sp. Cra20 changes the rhizosphere native bacterial community and promotes the growth of *Arabidopsis thaliana* by driving root developmental plasticity, thereby stimulating the growth of lateral roots and root hairs (Luo et al., 2019). Thokchom demonstrated that the inoculation with PGPR and plant age can affect bacterial communities in root tissues and rhizosphere soil (Thokchom et al., 2017).

Rhizosphere microorganisms are important because they can manage nutrient transformation, nutrient acquisition and use, and crop sustainability (Prasad et al., 2017). Rhizosphere microflora enhances the plant growth under abiotic stress through nitrogen fixation, plant hormone production, mineral solubilization, and iron carrier and HCN production. Furthermore, it activates the plant defense mechanisms against different bacterial and fungal pathogens (Mukhtar et al., 2019). Thus, the composition of rhizosphere–soil microbial communities plays an important role in the stability of plant, soil, and rhizosphere microbial communities (Lu et al., 2018). Further, syringic acid reportedly changes the community composition of bacteria and fungi in the cucumber rhizosphere, which may negatively impact the growth of cucumber seedlings by inhibiting the growth of plant-beneficial microorganisms (Wang et al., 2018). In contrast, PGPR can affect the microbial community succession and increase the plant yields (Zhang et al., 2019). Rhizosphere microorganisms play an important role in most ecosystem processes, and different crop management strategies applied to agricultural production can change the soil microbial composition. Further, VOCs produced by the beneficial microorganisms in the rhizosphere soil affect the rhizosphere microbial communities (Netzker et al., 2020). The present study shows that, in addition to inhibiting *M. incognita* infection, strain JF-22 changed the composition of the microbial community in the tomato rhizosphere and significantly altered the diversity of the fungal community. Such change was likely caused by volatile substances secreted by JF-22. However, the role of volatile organic compounds in the microbial community of strain JF-22 needs to be further studied to reveal the mechanism of strain JF-22-microbial community.

## Conclusion

This study used high-throughput and bioinformatics technology to study the effect of the JF-22 strain of *B. licheniformis* on the rhizosphere-soil microbial community and tomato plant growth. Strain JF-22 changed the composition of the microbial community in the tomato rhizosphere and significantly altered the diversity of the fungal community. Such change was likely caused by volatile substances secreted by JF-22. Using the FUNGuild and PICRUSt databases, we predicted the effect of JF-22 on microbial community function. Based on the demonstrated ability of JF-22 to control *M. incognita*, we believe that strain JF-22 of *B. licheniformis* can be considered a potential biocontrol agent for *M. incognita*.

## Data Availability Statement

The datasets presented in this study can be found in online repositories. The names of the repository/repositories and accession number(s) can be found below: NCBI SRA database, accession numbers PRJNA803317 and PRJNA803323.

## Author Contributions

XL, JD, and QG conceived and designed the experiment. XL, JD, QG, and CJ performed the experiment. XL, JD, QG, CJ, XS, YL, HL, CL, PZ, and JL analyzed the data. XL, JD, and QG wrote the manuscript. All authors read and approved the final manuscript.

## Conflict of Interest

The authors declare that the research was conducted in the absence of any commercial or financial relationships that could be construed as a potential conflict of interest.

## Publisher’s Note

All claims expressed in this article are solely those of the authors and do not necessarily represent those of their affiliated organizations, or those of the publisher, the editors and the reviewers. Any product that may be evaluated in this article, or claim that may be made by its manufacturer, is not guaranteed or endorsed by the publisher.

## Footnotes

1
http://drive5.com/uparse/
